# Supplementary material for: Improving the management and security of COVID 19 diagnostic test data with a digital platform in resource-limited settings: The case of PlaCARD in Cameroon
Source: PLOS Digit Health. 2022 Oct 5;1(10):e0000113. doi: 10.1371/journal.pdig.0000113 (PMC9931268; doi:10.1371/journal.pdig.0000113)
Supplement: Attachment — Submitted filename: Covid.docx [file pdig.0000113.s001.docx]

The authors of the manuscript “Improving the management and security of COVID 19 diagnostic test data with a digital platform in resource-limited settings: the case of PlaCARD in Cameroon.” Described the relevant topic.

They provided a lot of details about COVID 19 testing in Cameroon.

Improvements are necessary in this manuscript:

1. The entire manuscript is provided as a narrative, without clear separation of headings. Authors should re-write the manuscript and clearly divide Introduction, Methods, Results, etc.

2. Methodology section is more written as an introduction than standard methodology. The authors should provide methods that they used in the study as well as tests they used to determine the results of the COVID 19 testings. What statistics did they use to determine how significant are findings?

3. Results should provide at least some tables so readers can better understand the text provided in the results section.

4. Discussion should analyze obtained results and compare them with similar studies. That is lacking in this section.

5. Overall this is an interesting manuscript with the potential for improvements of the text, which is required before publishing.
