# Supplementary material for: Improving the management and security of COVID 19 diagnostic test data with a digital platform in resource-limited settings: The case of PlaCARD in Cameroon
Source: PLOS Digit Health. 2022 Oct 5;1(10):e0000113. doi: 10.1371/journal.pdig.0000113 (PMC9931268; doi:10.1371/journal.pdig.0000113)
Supplement: Attachment — Submitted filename: response to reviewers.docx [file pdig.0000113.s002.docx]

**Reviewer Comments**

Manuscript: PDIG-D-22-00034

Improving the management and security of COVID 19 diagnostic test data with a digital platform in resource-limited settings: the case of PlaCARD in Cameroon.

We thank the reviewers and the editor for pertinent comments that helped improve the quality of the manuscript. In what follows we address point-by-point r the various comments made by the editor and the reviewers.

**Journal Requirements:**

1. Please amend your detailed Financial Disclosure statement. This is published with the article. It must therefore be completed in full sentences and contain the exact wording you wish to be published.

State what role the funders took in the study. If the funders had no role in your study, please state: “The funders had no role in study design, data collection and analysis, decision to publish, or preparation of the manuscript.”

**As recommended by the editor, we have made the following change on financial disclosure: “The author(s) received no specific funding for this work.**

2. In the Funding Information you indicated that no funding was received. Please revise the Funding Information field to reflect funding received.

Please ensure that the funders and grant numbers match between the Financial Disclosure field and the Funding Information tab in your submission form. Note that the funders must be provided in the same order in both places as well.

**Indeed, no funding has been received for this study**

3. Please update your Competing Interests statement. If you have no competing interests to declare, please state: “The authors have declared that no competing interests exist.”

**As recommended by the editor, we have updated the interests statement as follow: “The authors have declared that no competing interests exist.”**

4. All figures and supporting information files will be published under the Creative Commons Attribution License (creativecommons.org/licenses/by/4.0/). Authors retain ownership of the copyright for their article and are responsible for third-party content used in the article.

Figure 2: please (a) provide a direct link to the base layer of the map used and ensure this is also included in the figure legend; (b) provide a link to the terms of use / license information for the base layer. We cannot publish proprietary or copyrighted maps (e.g. Google Maps, Mapquest) and the terms of use for your map base layer must be compatible with our CC-BY 4.0 license.

If your map was obtained from a copyrighted source please amend the figure so that the base map used is from an openly available source. Alternatively, please provide explicit written permission from the copyright holder granting you the right to publish the material under our CC-BY 4.0 license.

Please note that the following CC BY licenses are compatible with PLOS license: CC BY 4.0, CC BY 2.0 and CC BY 3.0, meanwhile such licenses as CC BY-ND 3.0 and others are not compatible due to additional restrictions.

If you are unsure whether you can use a map or not, please do reach out and we will be able to help you. The following websites are good examples of where you can source open access or public domain maps:

* U.S. Geological Survey (USGS) - All maps are in the public domain. (http://www.usgs.gov)

* PlaniGlobe - All maps are published under a Creative Commons license so please cite “PlaniGlobe, http://www.planiglobe.com, CC BY 2.0” in the image credit after the caption. (http://www.planiglobe.com/?lang=enl)

* Natural Earth - All maps are public domain. (http://www.naturalearthdata.com/about/terms-of-use/)

**We have updated Figure 4 by changing the base map from** [**http://maps.stamen.com/terrain/?request=GetCapabilities&service=WMTS&version=1.0.0#4/19.15/5.27**](http://maps.stamen.com/terrain/?request=GetCapabilities&service=WMTS&version=1.0.0#4/19.15/5.27) **which is licensed at CC BY 3.0. And the link to terms of use is** [**http://maps.stamen.com/#terrain/4/19.15/5.27**](http://maps.stamen.com/#terrain/4/19.15/5.27) **. We have provided the link to the base map and to the term of use web page in the revised manuscript. Line 281 to 285.**

Please upload any written confirmation as an 'Other' file type. It must clarify that the copyright holder understands and agrees to the terms of the CC BY 4.0 license; general permission forms that do not specify permission to publish under the CC BY 4.0 will not be accepted. Note that uploading an email confirmation is acceptable.

**Additional Editor Comments**:

Abstract: Kindly please divide into three sections (Background, Methodology/Principal Findings, and Conclusions/Significance).

**We have divided the abstract into three sections according to the editor's recommendations**

Methodology: Please revise and structure it accordingly. Needs to be more clear.

**As recommended by editor we have added a section in the methodology section that presents our main judgement criteria and the statistics we used. Lines 243 - 252.**

Discussion: Please revise the discussion. The Discussion should spell out the major conclusions of the work along with some explanation or speculation on the significance of these conclusions. How can future research build on these observations? What are the key experiments that must be done?

**As recommended by the editor we have revised the discussion section and added a paragraph in which we discuss our main results line 298 – 316.**

[Note: HTML markup is below. Please do not edit.]

**Reviewers' comments:**

Reviewer #1: The authors of the manuscript “Improving the management and security of COVID 19 diagnostic test data with a digital platform in resource-limited settings: the case of PlaCARD in Cameroon.” Described the relevant topic.

They provided a lot of details about COVID 19 testing in Cameroon.

Improvements are necessary in this manuscript:

1. The entire manuscript is provided as a narrative, without clear separation of headings. Authors should re-write the manuscript and clearly divide Introduction, Methods, Results, etc.

**We have revised the structure of the manuscript by clearly separating the heading of each section**.

2. Methodology section is more written as an introduction than standard methodology. The authors should provide methods that they used in the study as well as tests they used to determine the results of the COVID 19 testings. What statistics did they use to determine how significant are findings?

**As recommended by the reviewer we have added a section in the methodology section that presents our main judgement criteria and the statistics we used lines 243 - 252**

3. Results should provide at least some tables so readers can better understand the text provided in the results section.

**Since our results focus on two main criteria, namely the proportion of tests reported in PlaCARD and the turnaround time, we felt it was not necessary to put these results in a table**

4. Discussion should analyze obtained results and compare them with similar studies. That is lacking in this section.

**As recommended by the reviewer we have revised the discussion section and added a paragraph in which we discuss our main results line 298 – 316**

5. Overall this is an interesting manuscript with the potential for improvements of the text, which is required before publishing.

**We have gone through the text again to improve the quality and correct the typos as recommended by the reviewer.**

Reviewer #2: The authors described the architecture, implementation, and requirements of PlaCARD, a software platform to manage patient registration, medical specimens, diagnostic data flow, diagnosis results reporting, and authentication during the 2019 coronavirus pandemic (COVID -19) in Cameroon. The research work reported is of significant practice in the community.

Some suggestions are listed below to improve the manuscript’s quality.

1.	The representation in the introduction should be further improved, especially by highlighting the authors' contributions. The authors may consider some healthcare-based literature and cite them, e.g., 10.1016/j.knosys.2021.107338 and 10.1109/JIOT.2019.2949715.

**As recommended by the reviewer we have added a paragraph in the introduction to present the authors' contribution lines 119- 122.**

2.	The figures are a bit blurry. Please replace them with high-resolution pictures.

**We have replaced figures 2 and 4 by figures with a better resolution.**

3.	The authors may consider analyzing the in-depth reasons hidden in the experimental results.

**As recommended by the reviewer we have revised the discussion section and added a paragraph in which we discuss our main results line 298 – 316**

4.	In the conclusion, the future work should be discussed.

**A sentence was added in the discussion to introduce future work as recommended by the reviewer line 357- 360**

5.	There are some typos and grammar errors in the manuscript. Please polish the manuscript.

**We have gone through the text again to improve the quality and correct the typos as recommended by the reviewer**
